# Variation in Chlorophyll Content per Unit Leaf Area in Spring Wheat and Implications for Selection in Segregating Material

**DOI:** 10.1371/journal.pone.0092529

**Published:** 2014-03-27

**Authors:** John Hamblin, Katia Stefanova, Tefera Tolera Angessa

**Affiliations:** 1 Institute of Agriculture, University of Western Australia, Crawley, Western Australia, Australia; 2 School of Plant Biology, University of Western Australia, Crawley, Western Australia, Australia; ISA, Portugal

## Abstract

Reduced levels of leaf chlorophyll content per unit leaf area in crops may be of advantage in the search for higher yields. Possible reasons include better light distribution in the crop canopy and less photochemical damage to leaves absorbing more light energy than required for maximum photosynthesis. Reduced chlorophyll may also reduce the heat load at the top of canopy, reducing water requirements to cool leaves. Chloroplasts are nutrient rich and reducing their number may increase available nutrients for growth and development. To determine whether this hypothesis has any validity in spring wheat requires an understanding of genotypic differences in leaf chlorophyll content per unit area in diverse germplasm. This was measured with a SPAD 502 as SPAD units. The study was conducted in series of environments involving up to 28 genotypes, mainly spring wheat. In general, substantial and repeatable genotypic variation was observed. Consistent SPAD readings were recorded for different sampling positions on leaves, between different leaves on single plant, between different plants of the same genotype, and between different genotypes grown in the same or different environments. Plant nutrition affected SPAD units in nutrient poor environments. Wheat genotypes DBW 10 and Transfer were identified as having consistent and contrasting high and low average SPAD readings of 52 and 32 units, respectively, and a methodology to allow selection in segregating populations has been developed.

## Introduction

A recent review [1] considered that the rapid yield improvements of the last few decades were due primarily to increased Harvest Index of the economically important fraction of the crop with a more or less fixed biomass and/or from improved light harvesting through modified canopy structure. Further they suggested [1] that future increases in yield would have to come from improved photosynthetic efficiency. Several components of photosynthesis were examined for their potential in increasing crop yields. Amongst these was a reduction in leaf chlorophyll per unit area. There is little data on this issue partly because one of the most easily observed effects of N deficiency are pale green leaves, leading to the suggestion that using a SPAD 502 to measure leaf chlorophyll content per unit area could be a useful tool for determining N deficiency in crops [2], [3].

Despite the paucity of data, available evidence suggests that the relationship between yield and chlorophyll level per unit leaf area is worth further examination.

Working with a reduced chlorophyll mutant in Soybeans, it was found in some circumstances that the mutant biomass exceeded the wild type by 30% [4]. Crop yields of mutant rice lines were not constrained by reduced levels of chlorophyll [5]; [6]. Also grain yield increases in wheat over past decades were not due to an increase in the rate of photosynthesis [7], and no significant relationship was observed between photosynthetic rate per unit leaf area and chlorophyll content at high light intensities in a range of C4 plants including maize [8].

Hamblin [unpublished] using a variegated mutant of Arabidopsis, its wild type and the F1 found that chlorophyll per unit leaf weight was additive, but the effect on biomass showed over-dominance. The F1 was twice the weight of the wild type and 3 times the variegated mutant parent.

Two reasons were proposed as to why reduced chlorophyll levels might be beneficial [1]. First: less chlorophyll per unit leaf area would lead to improved light transmission though the canopy, potentially increasing photosynthesis of lower leaves and second: when exposed to excess light, more transparent leaves would reduce the level of photochemical damage to the chloroplasts in the upper canopy and thus less energy would be required for their repair.

The reason for excess chlorophyll in leaves above the optimum for photosynthesis may be that during evolution in the wild it was more important for survival to stop neighbours capturing light than it was to reduce photo-chemical damage by increased transmission through leaves [1]. However, this strategy is suboptimal for crops where productivity per unit area rather per plant drives improved production [1], [9].

Other possible benefits from reduced chlorophyll levels in leaves include (a) that because chloroplasts are nutrient rich, less chlorophyll per unit leaf area may “spare” nutrients that could be used for crop growth particularly in situations of sub-optimal nutrition and (b) reduced light capture above that needed for maximum photosynthesis may reduce the heat stress on leaves in the upper part of the canopy and less water may be needed for cooling, leaving more for grain filling.

The purpose of this study was (a) to determine within and between plant variation in leaf chlorophyll per unit leaf area in a range of environments, (b) identify genotypes that consistently differ in their levels of chlorophyll per unit leaf area and (c) obtain preliminary data on the impact of different environments on the ranking of genotypes.

Should genotypes differing consistently in chlorophyll per unit leaf area be identified, then a breeding programme, using a SPAD 502 can be developed to produce comparable lines that would allow testing of the hypothesis that lower levels of chlorophyll per unit leaf area improve crop yields.

## Materials and Methods

### Germplasm

Up to 26 wheat and 2 barley genotypes were used in a series of experiments to assess variation in chlorophyll per unit leaf area. These included between different positions on the leaf, between different leaves per plant, between plants, between genotypes and between environments to determine the impact of these factors on chlorophyll level per unit leaf area, measured with a SPAD 502. The details of the meter and underlying information on its use in chlorophyll measurement are available from the manufacturer [10].

The source, passport data and experimental coding of the genotypes used in each experiment are listed in Table 1. Limited seed was available for the 23 wheat and 1 barley genotypes obtained from the Australian Winter Cereals Collection (AWCC), Tamworth, NSW, Australia (now at Horsham, Victoria, Australia). Of the other 4 genotypes, W5; W6, and B2, (varieties grown in Western Australia) were obtained from InterGrain Pty Ltd and 1 genotype (W7) from Dr David Bowran.

**Table 1:** List of the genotypes used in the four experiments and obtained from the Australian Winter Cereals Collection (AWCC), InterGrain Pty Ltd (IGPL) and Dr. D. Bowran (DBW).

Genotype	Sources (AWCC No)	Exp Code	Exp 1	Exp 2	Exp 3	Exp 4
Wheat						
Ranee	AWCC (Aus1001)	W1	x	x	x	
Ranee (Variegated)	AWCC (Aus90113)	W2	x	x	x	
Alberta Red	AWCC (Aus1761)	W3	x	x	x	
Alberta Red (Variegated)	AWCC (Aus1762)	W4	x	x	x	
Emu Rock	IGPL	W5	x	x	x	x
Magenta	IGPL	W6	x	x	x	x
DBW 10	DBW	W7	x	x	x	
Kharchia	AWCC (Aus20741)	W8		x	x	
Janz	AWCC (Aus24794)	W9		x	x	
Stilletto	AWCC (AUS25923)	W10		x	x	
Pitic 62	AWCC (Aus804)	W11		x	x	
Tobari 66	AWCC (Aus1395)	W12		x	x	
Transfer	AWCC (Aus1406)	W13		x	x	x
Siete Cerros	AWCC (Aus1214)	W14			x	
Super X	AWCC (Aus6623)	W15			x	
Neepawa	AWCC (Aus12120)	W16			x	
Alfa	AWCC (Aus13900)	W17			x	
Uniculm 492	AWCC (Aus20430)	W18			x	
Oligoculm 112-76	AWCC (Aus20431)	W19			x	
CMH77A.917-1B-7Y-1B2Y-7B-0Y	AWCC (Aus21205)	W20			x	
Alfa	AWCC (Aus24324)	W21			x	
81W28-12	AWCC (Aus25186)	W22			x	
81W29-130	AWCC (Aus25192)	W23			x	
81W30-2	AWCC (Aus25194)	W24			x	
81W31-13	AWCC (Aus25209)	W25			x	
Excalibur	AWCC (Aus25292)	W26			x	
Barley						
BGS 306 va/3*Bowman (Variegated)	AWCC (Aus490473)	B1	x			
Bass	IGPL	B2	x			x

### Experimental conditions and treatments

Four experiments were conducted in the winter growing season of 2012 at the University of Western Australia's Field Station, Shenton Park, Western Australia. Experiment 3 was also conducted during the 2012/13 summer in a glasshouse at Shenton Park.

#### Experiment 1

Three seeds per pot of 9 genotypes (Table 1) were planted with 3 replications on 30.4.2012 in 15 cm pots in an evaporatively cooled glasshouse. All pots had 18 grams of Macrocote Purple slow release fertilizer and 6 grams of Hortico soil wetter granules. The main tiller of one plant per pot was tagged on germination and all measurements were made on these tillers.

Chlorophyll levels per unit area were estimated using a SPAD 502 on the youngest fully expanded leaf (YFEL1) and the second youngest fully expended leaf (YFEL2) on the tagged tillers. Measurements were made on five dates (04.06.12; 11.06.12; 22.06.12; 05.07.12; and 31.07.12). As time progressed and plants grew, the initial YFEL1 became YFEL2 and so on up the plant. All measurements were made between 10.00 and 12.00 a.m. The degree of cloud cover varied between and within dates. After each measurement date the pots were re-randomised.

Five evenly spaced SPAD readings, as judged by eye, were taken per leaf at each sample date, and overall 1350 measurements were made on the 27 tagged plants. These were recorded individually providing estimates of chlorophyll levels for YFEL1 and YFEL2 between different points on a leaf, between different leaves on a tiller, between different plants of the same genotype and between genotypes. Measurements stopped after the 5^th^ date when ear emergence occurred on the two earliest genotypes and these lines changed from vegetative to reproductive growth.

#### Experiment 2

This experiment was conducted with 13 wheat genotypes (Table 1) across 5 environments. The environments were 2 planting dates (30.04.2012 and 29.05.2012) each with two 2 fertiliser rates under field conditions. The two fertilizer rates were a low fertility environment with nil and a high fertility environment with 20 grams of Macrocote Purple slow release fertilizer applied per row at seeding.

The genotypes were sown in spaced East/West rows 50 cm long (approximately 10 seeds per row). Rows were 100 cm apart and 50 cm between bays. Rows were thinned at 4 weeks to 5 plants per row (per genotype). As seed was severely limited there was only one entry for each genotype in each environment. Although this causes problems with interpretation (see later discussion) it reflects breeding reality where effective selection should be as early as possible to reduce the amount of material requiring field testing in plots at commercial densities.

The fifth environment was planted on 02.07.2012 into 15 cm pots with 20 grams of Bunnings slow release complete fertilizer and 6 grams of Hortico soil wetter granules with 4 seeds per genotype and grown in an evaporatively cooled greenhouse.

Chlorophyll per unit area was estimated for each genotype with a SPAD 502. Measurements were made on 6 dates, 29.06.2012 (planting date 1 only), 23.07.2012, 07.08.2012, 23.08.2012 (all environments); on 29.08.2012 (planting date 2 and glasshouse) and on 14.09.2012 (glasshouse only). The first measuring date for any environment depended on whether the plants in that environment had reached a suitable size for measuring with SPAD 502 and the final date on whether any genotypes in that environment has changed from vegetative to reproductive growth. At each date 10 chlorophyll measurements per plot (genotype) were made on random YFELs in the field environments, while 5 measurements per pot were made on random YFELs in the glasshouse.

#### Experiment 3

After the first planting date of Experiment 2, a further 13 wheat genotypes were received (Table 1) and were included in the 2^nd^ planting date, high fertilizer treatment of experiment 2, which was planted on 29.05.2012 in the field and on 02.07.2012 in the glasshouse.

As in Experiment 2, chlorophyll per unit area was estimated with a SPAD 502. Measurements were made on 4 dates (26.07.2012; 07.08.2012; 23.08.2012; 29.08.2012) in the field and glasshouse environments. Additional measurements were taken on 13.09.2012 in the glasshouse environment only.

To provide an extreme alternative environment in terms of temperature and day length, the 26 wheat genotypes from Experiment 3 were planted on 30.1.2013 in an evaporatively cooled glasshouse and grown through February, the hottest month of the year in Perth, particularly so in 2013. Apart from the summer growing season, conditions were the same as those used in the glasshouse during the winter. SPAD readings were taken on the 24.02.2013 and 19.03.2013 when the earliest genotypes became reproductive.

#### Experiment 4

The SPAD 502 has been recommended for use in determining whether wheat crops are deficient in applied N and whether more should be applied [2]; [3]. Experiment 4 was established with 3 nutrient levels and 4 genotypes (Table 1) in pots in the glasshouse to assess the potential impact of fertility on SPAD units.

The 3 nutritional levels used were (1) potting compost with no added slow release fertilizer but with 100 ml every 10 days post-planting of Thrive nutrient solution, (2) Potting compost + 20 grams Bunnings slow release fertilizer and (3) Potting compost + 20 grams Bunnings slow release fertilizer plus 100 ml of Thrive nutrient solution every 10 days post-planting. All pots had 6 grams of Hortico Soil Wetter Granules.

Three of the genotypes used are currently grown by Western Australia farmers, two wheat genotypes (W5 and W6), one barley genotype (B2). W13 was also grown as it was consistently low in chlorophyll per unit area. Due to insufficient seed, W13 was planted in the high fertilizer treatment only. This was to test if improved fertility reduced the difference between W13 and the other genotypes. Five seeds per genotype per pot were planted on 03.07.2012 in 3 replications. The SPAD measurements were taken on 5 random YFELs on 30.08.2012 and 13.09.2012.

### Statistical analysis

Linear mixed models and ANOVA techniques were used to analyse the SPAD unit data generated from the four experiments varying in number of genotypes, replications and environments. All statistical analyses were performed using R (R Core Team, 2011) and GenStat, 15^th^ Edition (VSN International Ltd, UK, 2012).

#### Experiment 1

Chlorophyll levels were measured on the two youngest fully expanded leaves (YFEL1 and YFEL2) at 5 approximately evenly spaced points per leaf of 3 plants (replicates) for 9 varieties (7 wheats and 2 barley) over time. This is a typical repeated measurement experiment and as such the linear mixed model used to analyse data from this experiment accounted for possible correlations between the plants' measurements over time. The fixed part of the model comprised the main effects and the interaction of *Day* and *Variety*. The random part of the linear mixed model was specified by including terms *Plant+Plant.Day*. In addition, instead of assuming uniform correlations between the measurement dates over time, a more realistic model was employed assuming a decreasing correlation as time between measurements increases. This was done by fitting a first order auto-regressive model.

#### Experiments 2 and 3

These were conducted to assess variety chlorophyll rankings, and interactions over time. Due to seed shortages, these two experiments were not replicated. The SPAD measurements per genotype were averaged over all readings and the data was subjected only to exploratory data analysis (correlations and graphs).

#### Experiment 4

The experiment comprised 3 fertilizer treatments (F0, F1 and F2) applied to 4 genotypes Emu Rock (W5), Magenta (W6), Transfer (W13) and Bass (B2) planted in 3 replications. SPAD measurements taken were on 2 dates. Transfer was only planted in F2 fertiliser treatment due to seed limitations. As a result of the unbalanced data, the data was grouped into two fully balanced subsets and analysed separately. The first subset included 3 varieties by 3 fertiliser treatments. This data was analysed using a two-way ANOVA, where the *Variety* and *Treatment* main and interaction effects were fitted in the model along with *Date/Replication* blocking structure. The model was re-fitted after deleting outliers which did not alter the conclusions and will not be considered further. The second subset included four varieties and treatment F*2* only. This data was analysed using one-way ANOVA modelling only *Treatment* effect and included the same blocking structure.

## Results

### Experiment 1

**Figure 1:**
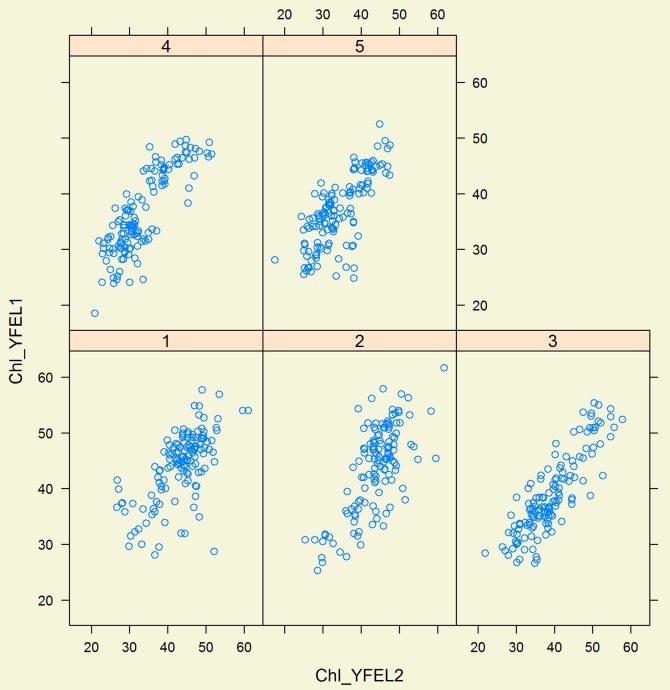
SPAD units (Chlorophyll) of first young fully expanded leaf (YFEL1) versus second young fully expanded leaf (YFEL2) for all five SPAD reading dates (1, 2, 3, 4 and 5) of experiment 1.

The SPAD unit measurements were highly consistent between measuring points on leaves and with the leaf mean within a date, between leaves on a tiller and over measuring dates. This is shown by the very good agreement of SPAD units between YFEL1 and YFEL2 for all genotypes and replications, for each of the 5 measurement dates (Fig. 1).

In all cases, the SPAD correlations were highly significant (p<0.001). The SPAD for date 5 YFEL1, (Table 2) illustrates the relationship between sampling positions on leaves over varieties and replicates on a given date. The variation accounted at each of the 5 recording dates/leaves correlations ranged from a low of 72% to a high of 92%.

**Table 2:** Correlations of date 5 YEFL (Young Fully Expanded Leaf) SPAD readings of chlorophyll content per unit area between 5 measurement positions and leaf mean in Experiment 1.

Position on leaf	2	3	4	5	leaf mean
1	0.87***	0.88***	0.83***	0.81***	0.92***
2		0.91***	0.85***	0.82***	0.94***
3			0.93***	0.92***	0.98***
4				0.97***	0.96***
5					0.95***

**Figure 2:**
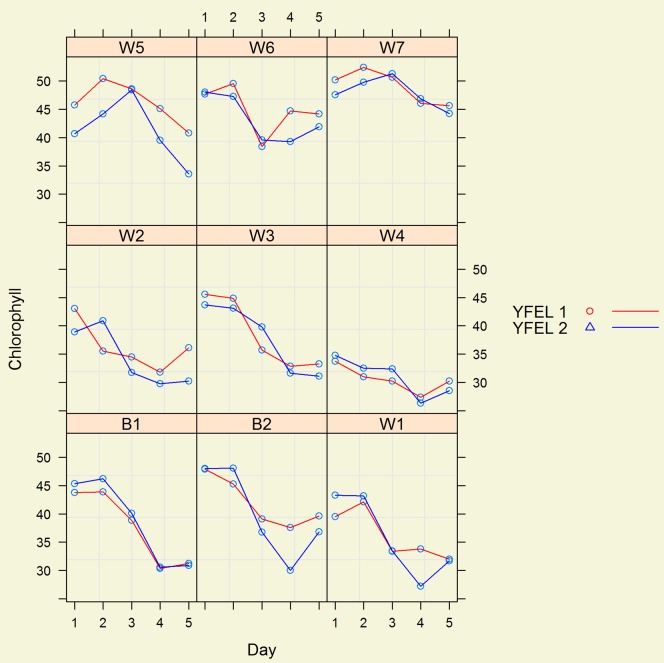
Declining pattern of SPAD units (chlorophyll) for the first young fully expanded leaf (YEFL1) and the second young fully expanded leaf (YEFL2) from the first through to the fifth (1, 2, 3, 4, 5) day of SPAD measurements for 7 wheat (W1–W7) and 2 barley (B1 and B2) genotypes in experiment 1.

When the SPAD data from experiment 1 was subjected to linear mixed model analysis, the main effects, of *Variety* and *Date of SPAD unit* by *Variety* interaction were highly significant (p<0.001). The SPAD units for both YFEL1 and YFEL2 for all varieties fell over time (Fig. 2).

Substantial and statistically significant genotypic differences were observed for the SPAD units as an indicator of chlorophyll content per unit area of leaf. The highest recording was from wheat genotype W7 (DBW 10) and the lowest reading was from wheat genotype W4 (Alberta Red Variegated) reflecting phenotypic variability of SPAD units, but attributable to genetic differences (Fig. 2, Table 3).

**Table 3:** Predicted means and SE for the chlorophyll content of YEFL1 and YEFL2.

Variety	Chlorophyll YFEL1	Chlorophyll YFEL2
B1	37.6	38.6
B2	41.9	40.0
W1	36.2	35.8
W2	36.2	34.3
W3	38.5	37.9
W4	30.5	30.9
W5	46.2	41.3
W6	44.9	43.2
W7	49.0	48.0
SE	0.46	0.60

### Experiment 2

**Figure 3:**
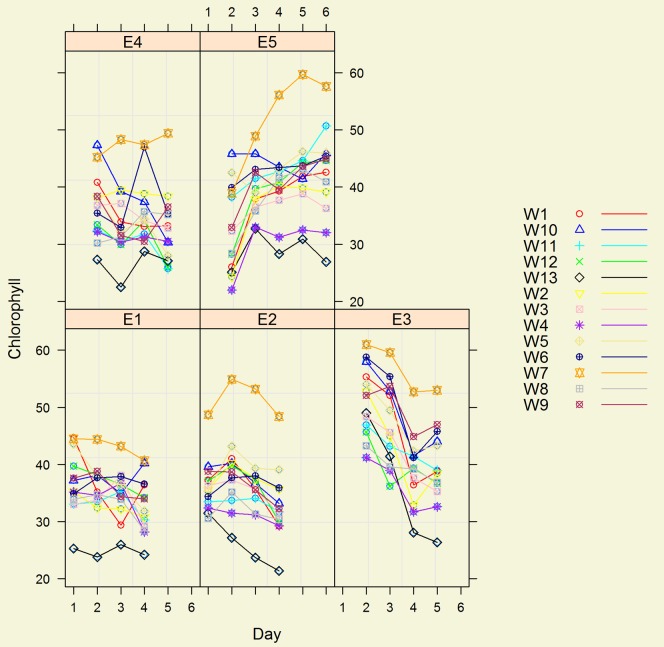
Wheat genotypes (W1–W13) SPAD units (Chlorophyll) in five environments (E1 Early sown High nutrition, E2 Early sown, Low nutrition, E3 Late sown, High nutrition, E4 Late sown low nutrition & E5 Glasshouse) taken on up to 5 days (1, 2, 3, 4, 5) in experiment 2.

The SPAD 502 can automatically calculate the means of a series of readings. Ten measurements were made per YFEL of each genotype in each environment in the field and 5 in the glasshouse. These were averaged and the mean recorded as a genotype's SPAD value. The pattern of genotypic response to different environments changed with the environment (Fig. 3). In environments E1, E2 and E4, only slight changes in SPAD units occurred over time; but there was a marked decrease of SPAD units in environment E3 and a marked increase in environment E5 (Fig. 3). Environment E5 was conducted in the glasshouse and its SPAD pattern over time was the opposite of that found in Experiment 1, which was also grown in the glasshouse but planted some 2 months earlier where for both YFEL1 and YFEL2, the SPAD units predominantly fell with time (Fig. 2). Wheat genotype W7 recorded the highest SPAD units in experiments 1 and 2 (Figs. 2 and 3).

Correlation analysis revealed that SPAD units between genotypes in different environments were highly and significantly correlated p<0.001 (Table 4). In terms of an individual genotype's SPAD units as an indicator of chlorophyll content per unit leaf area, wheat genotype W7 had the highest level (50.3) and genotype W13 had the lowest (28.5) averaged across all five environments.

**Table 4:** Correlation matrix of rankings of mean SPAD readings over dates in Experiment 2 for all genotypes and environments (E1–E4) in the field arising from combination of 2 planting dates (DOP) and 2 fertility levels (Low and High) and in the glasshouse (GH) as the fifth environment (E5).

	DOP	01.05.12	29.05.12	29.05.12	02.07.12	
DOP	Fertility	Low	High	Low	GH	Mean
**01.05.12 (E1)**	**High**	0.89***	0.80***	0.76***	0.83***	0.91***
**01.05.12 (E2)**	**Low**		0.85***	0.89***	0.85***	0.96***
**29.05.12 (E3)**	**High**			0.82***	0.88***	0.94***
**29.05.12 (E4)**	**Low**				0.71**	0.90***
**GH (E5)**						0.93***

### Experiment 3

**Figure 4:**
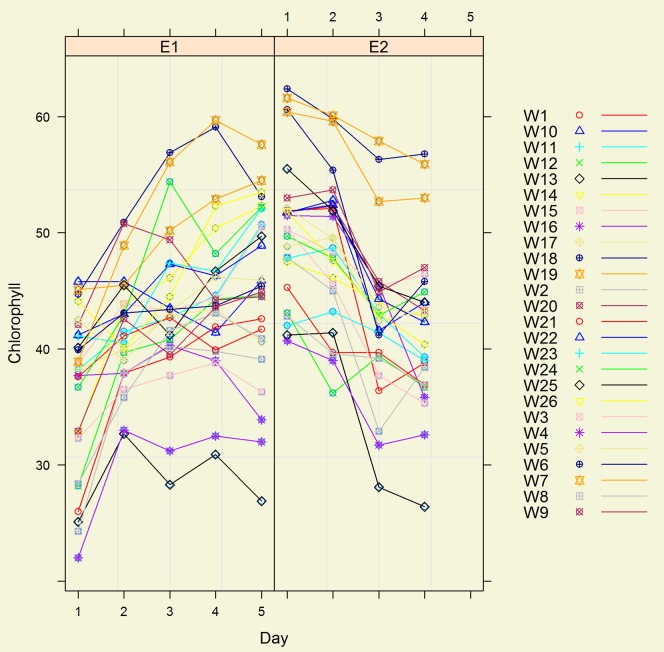
Wheat genotypes (W1–W26) SPAD units (Chlorophyll) in environments E1 (field based high fertility) and E2 (winter glasshouse) taken on 5 days in experiment 3.

Two distinctive patterns were observed in environments 1 and 2 over time. There was a steady increase in SPAD units over time in environment 1 for all genotypes whereas in Environment 2 there was a decrease (Fig. 4).

Despite different patterns of response over time for the genotypes in the different environments (Winter field, winter glasshouse and summer glasshouse), the correlations were remarkably stable not only between the 2 winter environments (Fig. 4) but also with the summer ranking of varieties for chlorophyll content. The correlation between winter field and winter glasshouse environments was (r = 0.84*** significant at p≤0.001), between winter glasshouse and summer glasshouse was (r = 0.60** significant at p≤0.01), and between winter field and summer glasshouse was (r = 0.51** significant at p≤0.01).

### Experiment 4

Due to the unbalanced nature of the design of this experiment, two separate analyses were performed. The first analysis involved 3 genotypes, Emu Rock (W5) Magenta (W6) and Bass (B2), and 3 fertiliser treatments (F0, F1 and F2). The treatment effect was highly significant (p<0.001), whilst the genotype effect was not significant (p = 0.131) Table 5. The genotype by treatment interaction was significant (p = 0.002). Genotypic means were very similar under fertiliser treatments F1 and F2 but significantly different in treatment F0. Within treatment F0, barley (Bass, B2) had a significantly higher SPAD reading than wheat at the first SPAD reading date (Table 5). This was because barley was initially much more vigorous than wheat in treatment F0.

**Table 5:** Mean SPAD units of chlorophyll content per unit leaf area of wheat genotypes Emu Rock, Magenta and Transfer and barley genotype Bass assessed under fertiliser treatments F0, F1 and F2 in Experiment 4.

Treatment/Genotype	Bass	Emu Rock	Magenta	Transfer
F0	32.4	19.8	24.1	N.T.
F1	47.1	49.1	46.2	N.T.
F2	48.1	49.8	50.0	33.9
a/SED	2.6			N.A.
a/LSD (0.05)	5.3			N.A.
b/SED	1.9			
b/LSD (0.05)	4.0			

a/ indicates SED or LSD when 3 genotypes tested in all 3 fertiliser treatments;

b/ indicates SED or LSD when all 4 genotypes tested in only F2 fertiliser treatment);

N.T. - Not Tested;

N.A. - Not Applicable.

The second analysis involved treatment F2 only, where the wheat genotype Transfer was tested alongside three other genotypes (Table 5). The result revealed that the genotypic effect was highly significant (p<0.001). This was due to Transfer's SPAD reading being substantially less than the other 3 genotypes in fertiliser treatment F2 (Table 5).

## Discussion

SPAD units as estimates of leaf chlorophyll content per unit area from different points on a leaf and from different leaves of the same plant were highly correlated to SPAD units of other leaves on the same plant in Experiment 1 (Fig. 1; Table 2). Differences between genotypes for SPAD readings were large. From a plant selection perspective, a series of measurements using a SPAD 502 on a single leaf and averaging the measurements provides an adequate ranking assessment of genotypes for their level of leaf chlorophyll per unit area. Averaging several measurements to obtain a mean improves selection efficiency and reduces measurement and recording times and costs [11].

Despite the fact that there was only one entry per genotype in the different environments of Experiments 2 and 3, and there were easily observable interactions between genotypes and environments (Figs. 3, 4), nonetheless the ranking of varieties between environments were remarkably similar in both experiments (Table 4 and results section Experiment 3). This consistency was independent of the time of measurement and leaf position when the measurements were taken (Tables 2, 4). Such high consistency in genotype ranking was unexpected as there are reported to be at least 17 additive and 9 epistatic QTLs for chlorophyll content in wheat and that 10 of the additive QTLs are expressed at different growth stages [12]. In this circumstance a large G x E interaction in genotype rankings and no significant correlations might be expected but were not observed. The results show that it should be possible to select for genotypic differences in levels of chlorophyll content per unit leaf area in spring wheat either on single plants or in short rows at one time using a SPAD 502. This is in agreement with the observation that SPAD chlorophyll measurements have intermediate heritability and are of use in selection [7].

Unlike all the other genotypes used with stable SPAD units across environments, wheat genotype W4 (Variegated Alberta Red) produced seedlings that were variegated, albino (up to 40%) and green. Although this genotype had consistently low mean SPAD readings of 32 units across all environments, its unstable leaf variegation indicates that W4 is likely to be a chloroplast mutant [13] and not suitable for a breeding program aimed examining the hypothesis that less chlorophyll may be a path to higher yields.

From a practical point of view, SPAD measurements should be taken from fully expanded young upper leaves. When genotypes differing in days to flowering are studied, measurements should be taken before flowering of the earliest genotypes or genotypes should be grouped according to maturity before SPAD measurements are taken.

Results for the 3 modern genotypes, Emu rock, Magenta and Bass, grown at three fertiliser levels showed significant genotypes by fertiliser interactions in the F0 treatment as compared to the F1 and F2 treatments. In the no fertiliser (F0) environment, the barley genotype, Bass, had more vigorous growth and a higher SPAD reading at the first measurement date, where all genotypes had low SPAD readings (Table 5). Under environments with fertilisers (F1 and F2), wheat genotypes improved and their SPAD units were the same as those of barley. Despite the high nutrition of the F2 environment, W13 (Transfer) was significantly lower in SPAD units than the other wheat genotypes.

The SPAD 502 has been recommended for use in determining whether wheat crops are deficient in N [2], [3]). Results from experiment 4, which was conducted to establish the impact of soil fertility on the level of SPAD units showed that the wheat genotype W13 maintained its significantly lower SPAD readings at high levels of plant nutrition. This result was in agreement with observations in experiments 2 and 3 where W13 always had low SPAD readings.

Two wheat genotypes, W7 (DBW 10) with high mean SPAD units of 52 and W13 (Transfer) with low mean SPAD units of 32 have been identified as having consistent and contrasting levels of leaf chlorophyll content per unit area.

## Future Prospects

The substantial and consistent differences in SPAD units between W7 and W13 will be the basis of future agronomic, breeding, genetic, and mapping studies of chlorophyll content in spring wheat. Together with two commercial wheat cultivars (Emu Rock and Magenta with intermediate SPAD units) they have been crossed to develop pure lines to be used to determine the effect of different chlorophyll levels on yield in spring wheat.
